# Reversal of Diabetic Nephropathy by a Ketogenic Diet

**DOI:** 10.1371/journal.pone.0018604

**Published:** 2011-04-20

**Authors:** Michal M. Poplawski, Jason W. Mastaitis, Fumiko Isoda, Fabrizio Grosjean, Feng Zheng, Charles V. Mobbs

**Affiliations:** 1 Fishberg Center for Neurobiology, Mount Sinai School of Medicine, New York, New York, United States of America; 2 Department of Internal Medicine, Yale University School of Medicine, New Haven, Connecticut, United States of America; 3 Department of Geriatrics and Palliative Medicine, Mount Sinai School of Medicine, New York, New York, United States of America; Pennington Biomedical Research Center, United States of America

## Abstract

Intensive insulin therapy and protein restriction delay the development of nephropathy in a variety of conditions, but few interventions are known to reverse nephropathy. Having recently observed that the ketone 3-beta-hydroxybutyric acid (3-OHB) reduces molecular responses to glucose, we hypothesized that a ketogenic diet, which produces prolonged elevation of 3-OHB, may reverse pathological processes caused by diabetes. To address this hypothesis, we assessed if prolonged maintenance on a ketogenic diet would reverse nephropathy produced by diabetes. In mouse models for both Type 1 (Akita) and Type 2 (db/db) diabetes, diabetic nephropathy (as indicated by albuminuria) was allowed to develop, then half the mice were switched to a ketogenic diet. After 8 weeks on the diet, mice were sacrificed to assess gene expression and histology. Diabetic nephropathy, as indicated by albumin/creatinine ratios as well as expression of stress-induced genes, was completely reversed by 2 months maintenance on a ketogenic diet. However, histological evidence of nephropathy was only partly reversed. These studies demonstrate that diabetic nephropathy can be reversed by a relatively simple dietary intervention. Whether reduced glucose metabolism mediates the protective effects of the ketogenic diet remains to be determined.

## Introduction

While intensive insulin therapy and other interventions slow the development of diabetic complications [1], there is far less evidence that these interventions reverse diabetic complications. For example, tight glucose control prevented the development of nephropathy (as indicated by proteinuria) in a rat model of Type 1 diabetes, but did not reverse nephropathy once proteinuria had developed [2]. Thus there is a general consensus that diabetes is associated with progressive and cumulative processes that are much more amenable to retardation than to reversal. Nevertheless, from a clinical perspective, reversing pathologies associated with diabetes would be far more valuable than simply delaying their onset.

We have proposed that both diabetic complications and age-related pathologies develop due to a progressive and cumulative effect of glucose metabolism that produces a bistable hysteretic effect on gene expression [3]. In addition to glycolytic enzymes that would be expected to produce oxidative stress [3], glucose metabolism also induces a variety of molecular responses such as thioredoxin-interaction protein [4] and p65 [5] that could plausibly contribute to nephropathy. Indeed, the latter induction is persistent, even after normalization of glucose, thus exemplifying glucose-induced hysteresis and its clinical correlate, metabolic memory, including in nephropathy [6]. Furthermore, based particularly on detailed analysis of the hysteretic behavior of the lac operon [7], [8], we have hypothesized that sufficiently prolonged and robust reduction in glucose metabolism or molecular responses to glucose metabolism may reverse this bistable molecular state, leading to reversal of pathology [3]. While examining basic mechanisms mediating molecular responses to glucose, we observed that the ketone 3-beta-hydroxybutyric acid (3-OHB) blocked the inhibition of agouti-related peptide by glucose [9]. Together with evidence suggesting that the ketogenic diet produces a prolonged exposure to 3-OHB, these data suggest that the ketogenic diet may prevent and even reverse pathologies associated with diabetes. One manipulation that produces a chronic elevation in 3-OHB levels is caloric restriction, and we have hypothesized that this elevation in 3-OHB may mediate many of the protective effects of dietary restriction [3]. On the other hand, chronic caloric restriction is not a plausible intervention for clinical use. An alternative approach to producing chronic elevation of 3-OHB is a ketogenic diet, used clinically to treat refractory epilepsy [10]. Although the mechanism by which the ketogenic diet reduces the frequency of epileptic seizures is not entirely understood, most evidence suggests that it does so by reducing neuronal glucose metabolism [11], [12]. Furthermore, analysis of the effect of the ketogenic diet on gene expression is consistent with the hypothesis that the ketogenic diet does re-route cellular metabolism away from glucose utilization and toward the use of alternative fuels, presumably including 3-OHB [13], [14]. In the present studies we therefore examined if a ketogenic diet would reverse pathologies produced by diabetes. We focused on nephropathy because kidney function is relatively amenable to repetitive assessment simply by repetitive collection of urine, thus allowing definitive determination that pathology had developed before beginning the dietary intervention. These studies clearly demonstrate that diet can reverse diabetic kidney disease even after it has developed.

## Methods

### Ethics Statement

All studies were approved by the appropriate institutional animal review board (Institutional Animal Care and Use Committee, IACUC, Mount Sinai School of Medicine; Approval ID 07-0044, “Reversal of Diabetic Complications by Diet”). All animals used in these studies were obtained from The Jackson Laboratory (Bar Harbor, ME) and housed with free access to food and water under 12:12-h light-dark cycle (lights on at 7:00 A.M.).

### Akita diabetic nephropathy

Male 10-week old C57Bl/6J mice (wild-type or with the Akita mutation (JAX#003548, C57BL6-*Ins2akita*/J) (n = 28 for each genotype) were purchased from Jackson Laboratories. At 20 weeks of age urine was collected for urinalysis, and development of diabetic nephropathy was confirmed by elevated albumin-to-creatinine ratio (ACR). At that time, half of wild-type mice and half of the Akita mice were placed on a ketogenic diet (5% carbohydrate, 8% protein, 87% fat) (#F3666, BioServ, Frenchtown, NJ). The remaining animals were maintained on a standard AIN-93M-based high-carbohydrate control diet (64% carbohydrate, 23% protein, 11% fat). Mice were maintained on the ketogenic diets for 8 weeks at which time urine was collected for determination of ACR, followed by sacrifice using a balanced design at the start of the light period (10:00 A.M. to 2:00 P.M.). Mice were sacrificed by decapitation after a brief exposure to carbon dioxide. However, within 2 weeks after switching mice to the ketogenic diet, 2 mice on the chow diet had died, whereas none of the mice on the ketogenic diet had died, raising the possibility that by 8 weeks there would be too few chow control mice left to allow direct comparison of gene expression. Therefore all mice on the chow control diet were sacrificed 2 weeks after switching the experimental mice to the ketogenic diet, 6 weeks before those mice were sacrificed. No mice on the ketogenic diet died spontaneously during the course of the study. Kidneys were collected and fresh-frozen for analysis of gene expression.

### *db/db* diabetic nephropathy

Male 10-wk old C57Bl/KsJ (wild-type or *db/db*; JAX#000642, BKS.Cg-*Dock7^m^*+/+*Leprdb*/J) (n = 20 each genotype) were purchased from Jackson Laboratories. At 12 weeks of age half of each genotype were placed on a ketogenic diet (5% carbohydrate, 8% protein, 87% fat). The remaining animals were maintained on a standard AIN-93M-based high-carbohydrate control diet (64% carbohydrate, 23% protein, 11% fat). Urine was collected after 8 weeks on the diet in all groups. Body weight, blood glucose, and blood ketone levels were monitored throughout the study. After 8 weeks on the diet, all animals were sacrificed following a balanced design at the start of the light period (10:00 A.M. to 2:00 P.M.). Mice were killed by decapitation after a brief exposure to carbon dioxide. Kidneys were collected and one was fresh-frozen for gene expression analysis, and the other fixed for renal histopathology.

### Measurement of UAE

Excretion of urinary albumin was determined using albumin-to-creatinine ratio (ACR) in 24 hr urine collections. Twenty-four-hour urine was collected using Nalgene® Metabolic Cage System (Rochester, NY). Nalgene metabolic cages are designed to allow efficient separation of the urine and feces from a single mouse. The concentration of creatinine in urine was determined using The Creatinine Companion kit (Exocell, Philadelphia, PA) and that of albumin using Albuwell M kit (Exocell).

### Glomerular Morphometry

At time of sacrifice, kidneys were harvested for pathological examination. One kidney was fresh-frozen for gene expression analysis, and the other was fixed in 10% neutral buffered formalin (Sigma-Aldrich Co., St. Louis, MO). The formalin-fixed tissue was embedded in paraffin and 4-µm sections were stained with periodic acid-Schiff stain (American Histolabs, Gaithersburg, MD). Glomerular sclerosis was quantified using a semi-automatic image analysis technique with examination of the total cortical area. To perform the morphologic analysis, a total of 30 glomeruli were randomly selected from each kidney moving the slide from the outer to the inner cortex in a random fashion to obtain non-overlapping sample fields. Glomerular images were recorded using a CCD camera (Sony, Tokyo, Japan) mounted on light microscope (Zeiss, Gottingen, Germany). Glomerular tuft surface area was obtained using the MetaMorph image analysis computer program (Universal Imaging Co., West Chester, PA). From the glomerular tuft image, the amount of periodic acid– Schiff–positive material was selected automatically by use of the color recognition properties of the software. The number of pixels in this area was considered to represent the area of sclerosis and was expressed as a fraction of the tuft surface area.

### *In vitro* assessment of cytoprotective effects of the ketone 3-beta-hydroxybutyrate (3-OHB)

To directly assess if the ketone 3-OHB is cytoprotective, we adapted a basic assay for cellular sensitivity to oxidative stress [15], [16], [17], [18], [19], [20], and further validated the protocol for the modulation of oxidative stress by glucose. Primary cortical neurons were prepared from embryonic (E16) brains using standard methods [21], [22]. Neurons were maintained at 20 mM glucose in Neurobasal/B27 medium (Invitrogen) for 5 days to allow neuronal expansion to the appropriate density. Cells were then exposed to various concentrations of 3-OHB and/or glucose for 2.5 hours, then exposed to 100 uM hydrogen peroxide for one hour (controls were simply exposed to a change in medium without hydrogen peroxide). After one hour the medium was removed, wells are washed three times with fresh medium (all wells now at 20 mM glucose). Twenty-four hours later, cellular viability was assessed using the CKK-8 assay (Dojindo Molecular Technologies).

### Extraction of kidney RNA and cDNA synthesis

To obtain RNA for gene expression analysis by quantitative PCR, renal tissue was homogenized in tubes containing RLT buffer (Qiagen) supplemented with 2-ME, and total RNA was extracted using an RNeasy Mini Kit (Qiagen). Total RNA was quantified using a Biophotometer (Eppendorf). Due to capacity limitations of the PCR array plates, four to five samples from each experimental group were selected (based on superior RNA quality) and were subjected to reverse transcription. 1 ug of high-quality total RNA was used for cDNA synthesis using RT^2^ First Strand Kit (SAbiosciences). All procedures were performed according to the manufacturers' protocols.

### RT-PCR with RT^2^ Profiler™ PCR Arrays

RT^2^ Profiler PCR Array (SuperArray Bioscience Corporation) technology for gene expression analysis entails a synthesis between the profiling capabilities of DNA microarray and the quantitative reliability and sensitivity of quantitative PCR. The results are highly reproducible within the same assay run or between different assay runs. Two cataloged RT^2^ Profiler PCR Arrays were used to simultaneously quantify mRNAs of 160 genes (PAMM-065 – Oxidative Stress and Antioxidant Defense PCR Array, PAMM-003 – Stress & Toxicity PCR array), including five “housekeeping genes” in 384-well plates according to the protocol of the manufacturer (SuperArray Bioscience). The qPCR reactions were carried out using an ABI Prism 7900 thermocycler. Average expression of the five “housekeeping genes” on the array were used to normalize the gene expression by the ΔΔCt method. Data were analyzed using a web-based software program provided by the manufacturer with additional analysis using GraphPad Prism 4 for Macintosh.

### Additional RT-PCR

Quantitative PCR for three genes – nephrin, podocin and ZO-1 (Tjp1, tight junction protein 1) – was carried out on separate 384-well plates using primers purchased from SABiosciences and normalized to expression levels of Rpl13a (Ribosomal protein L13a). The qPCR reactions were carried out using an ABI Prism 7900 thermocycler with conditions specified by SABiosciences for their RT^2^ qPCR Primer Assays.

### Blood chemistry

Blood glucose was measured by a Bayer Contour glucose meter (Bayer, Mountain View, CA). Blood 3-OHB was measure by a Precision Xtra Ketone Monitoring System (Abbott Laboratories. Abbott Park, IL).

### Data Analysis

All data are presented as mean ± SEM. Statistical analysis was performed using GraphPad Prism 4.0 by one-way ANOVA followed by Newman-Keul's post hoc test. P<0.05 indicates statistical significance.

## Results

### Reduction of blood glucose and increase in blood 3-OHB by ketogenic diet

As expected, the ketogenic diet produced elevated blood 3-OHB in all groups, particularly in the diabetic mice (Table 1). Furthermore, after 2 months on the ketogenic diet blood glucose was reduced in wild-type mice, completely normalized in Akita (Type 1) and partly normalized in db/db (Type 2) diabetic mice. The ketogenic diet also reduced body weight in wild-type mice but not in diabetic mice, even though the diet reduced caloric intake in db/db mice. None of the conditions influenced blood pH, although there was a non-significant trend toward reduced blood pH (i.e., acidification) in diabetic mice, and the ketogenic diet reversed this trend.

**Table 1:** Physiological characteristics.

	Body Weight (g)	Blood Glucose (mg/dl)	Blood 3-OHB (mmol/L)
Wt-Chow	33.7±0.75	134.6±10.2	0.15±0.02
Wt-Keto	29.6±0.67†	97.3±5.3†	0.63±0.1†
Akita-Chow	21.2±1.25†	441.9±39†	0.47±0.1†
Akita-Keto	23.0±0.68†	113.6±8.5	2.34±0.3†
Wt-Chow	26.2±0.37	125.8±6.3	0.2±0.02
Wt-Keto	23.8±0.81†	90.9±5.2†	1.1±0.2†
db/db-Chow	39.6±1.88†	601±0.0†	0.4±0.05†
db/db-Keto	54.5±1.53†	299±54.5†	1.8±0.5†

†p<0.05 compared to respective Wt-Chow groups.

### Reversal of albuminuria in diabetic mice

**Figure 1:**
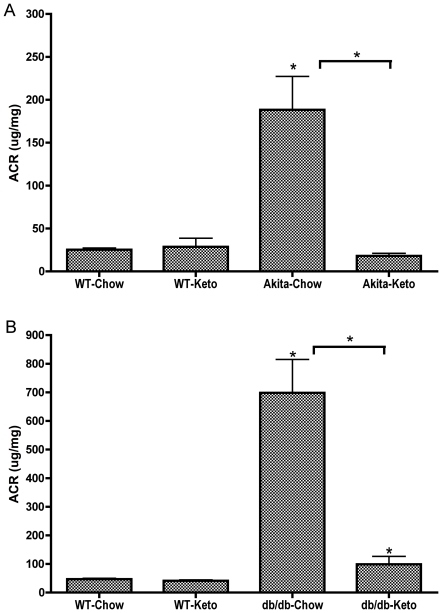
Development of albuminuria in mouse models of type 1 and type 2 diabetes and its reversal by the ketogenic diet. ACR in diabetic Akita mice (A) and in diabetic *db/db* mice (B) with respective age-matched control mice, fed either the ketogenic diet or chow diet for 8 weeks. Urine was collected and ACR measured 8 weeks after starting the diet regimens. Data are means ± SE (n = 10–14 for all groups). *p<0.05 (ANOVA) vs. WT-Chow group (or vs. diabetic-Chow groups as indicated by the horizontal brackets).

As anticipated [23] Akita mice developed hyperglycemia around 4 weeks of age and by 20 weeks of age Akita mice had all developed nephropathy on the control diet, as indicated by a roughly 10-fold increase in urinary albumin/creatinine ratios (Figure 1A). At that point half of the diabetic Akita mice and half of the euglycemic mice were placed on the ketogenic diet, whereas half of the diabetic and half of the euglycemic mice were continued on the control (normal high-carbohydrate) diet. Within one week after switching to the ketogenic diet, blood glucose in Akita mice was completely normalized (127+/−17 mg/dl), without insulin treatment; blood glucose fell slightly in the euglycemic mice (83+/−8 mg/dl) on the ketogenic diet. Within 2 weeks, 4 of the Akita mice on the control diet had died, reducing the n to 8, whereas none of the mice on the ketogenic diet had died. Since it was clear that all the control Akita mice would be dead within 2 months (consistent with other survival curves of mice with Type 1 diabetes [24]), when we anticipated the Akita mice would be sacrificed, thus preventing post-sacrifice comparison of kidney oxidative stress and histology, we elected to sacrifice all mice on the control diet (wild-type and Akita), and to continue to monitor blood glucose and urinary albumin/creatinine ratios in mice on the ketogenic diet. Remarkably, within 2 months, diabetic nephropathy was completely reversed as indicated by urinary albumin/creatinine ratios (Figure 1A). Figure 1A shows just the data from the group of Akita mice that was placed on the ketogenic diet, for clarity. “Akita-Chow” actually refers to the group of Akita mice that were on chow up until 20 weeks, then switched to the ketogenic diet. “Akita-Keto” refers to the same mice at 28 weeks, after having been on the ketogenic diet for 8 weeks. The ketogenic diet had no effect on albumin/creatinine ratios in euglycemic control mice. The key observation is that after 8 weeks on the ketogenic diet, the formerly “Akita/Chow” mice exhibited complete reversal of diabetic nephropathy, as demonstrated by albumin/creatinine ratios. Similarly, db/db mice developed hyperglycemia by 12 weeks of age, at which time half of the mice were switched to the ketogenic diet. In contrast to Akita mice, the ketogenic diet did not completely normalize blood glucose in db/db mice, but did reduce blood glucose by about 50%. Nevertheless, within 8 weeks of switching to the ketogenic diet, albuminurea was almost completely corrected (Figure 1B).

### Reversal of stress- and toxicity-related gene expression induced with Type 1 and Type 2 diabetes in kidney

**Figure 2:**
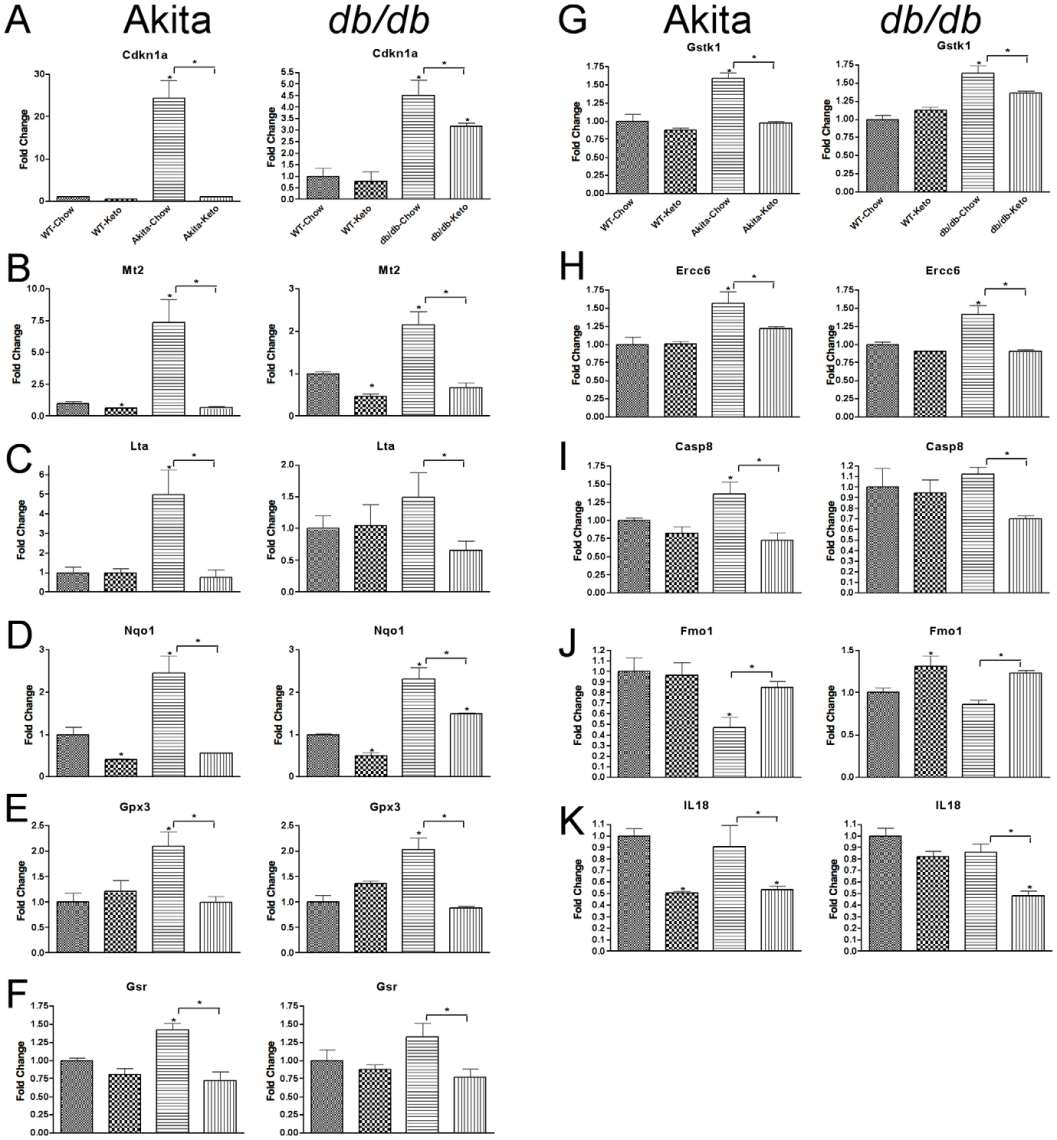
Diabetic nephropathy is associated with a similar profile of gene expression for oxidative stress and toxicity related transcripts in models of Type 1 (Akita) and Type 2 (*db/db*) diabetes – reversal with ketogenic diet. Real-time PCR gene expression was assessed by RT^2^ Profiler PCR Arrays from SABiosciences. The ketogenic diet reverses the kidney gene expression profile associated with diabetic nephropathy in both the Akita and *db/db* mice. Data for each gene was normalized to a panel of housekeeping transcripts and expressed as fold change compared to the WT-Chow group. Data are means ± SE (n = 4–5 for all groups). *p<0.05 (ANOVA) vs. WT-Chow group (or vs. diabetic-Chow groups as indicated by the horizontal brackets).

In view of the functional reversal of diabetic nephropathy by the ketogenic diet, we examined if the functional reversal was accompanied by reversal of molecular evidence of diabetes-induced toxicity. To carry out this assessment we examined expression of genes implicated in response to cellular stress, particularly oxidative stress, using quantitative PCR arrays (SABiosciences). This analysis led us to discover 9 genes whose expression in kidney is induced both in Type 1 and in Type 2 diabetes (Figure 2; Table 2). In general the induction of these genes was more robust in Akita than in db/db mice. Of particular interest, the elevated expression of every gene was completely reversed by the ketogenic diet in Akita mice and largely or completely reversed in db/db mice, which is remarkably consistent with effects on albuminuria. One gene, Fmo1, was inhibited in both Type 1 and Type 2 diabetes (Figure 2J), and this inhibition was completely reversed in both conditions by the ketogenic diet. IL-18 (Figure 2K) exhibited yet a different pattern, in that its expression was not influenced by diabetes but was inhibited by the ketogenic diet, which could thereby produce some protective effect directly.

**Table 2:** List of genes whose expression is described in the manuscript.

Symbol	GeneID	Gene Name
Casp8	12370	Caspase 8
Cdkn1a	12575	Cyclin-dependent kinase inhibitor 1A (P21)
Duox1	99439	Dual oxidase 1
Ercc6	319955	Excision repair cross-complementing rodent repair deficiency, complementation group 6
Fmo1	14261	Flavin containing monooxygenase 1
Gpx2	14776	Glutathione peroxidase 2
Gpx3	14778	Glutathione peroxidase 3
Gsr	14782	Glutathione reductase
Gstk1	76263	Glutathione S-transferase kappa 1
Hmox1	15368	Heme oxygenase (decycling) 1
Hmox2	15369	Heme oxygenase (decycling) 2
IL18	16173	Interleukin 18
Lta	16992	Lymphotoxin A (TNF-beta)
Mt2	17750	Metallothionein 2
Nephrin	54631	nephrosis 1 homolog (human), Nphs1
Nos2	18126	Nitric oxide synthase 2, inducible
Nox1	237038	NADPH oxidase 1
Nox4	50490	NADPH oxidase 4
Nqo1	18104	NAD(P)H dehydrogenase, quinone 1
Podocin	170484	nephrosis 2 homolog (human), Nphs2
Sod1	20655	Superoxide dismutase 1, soluble
Txnip	56338	Thioredoxin interacting protein
ZO-1	21872	Zonula occludens 1, tight junction protein 1 (tjp1)

**Figure 3:**
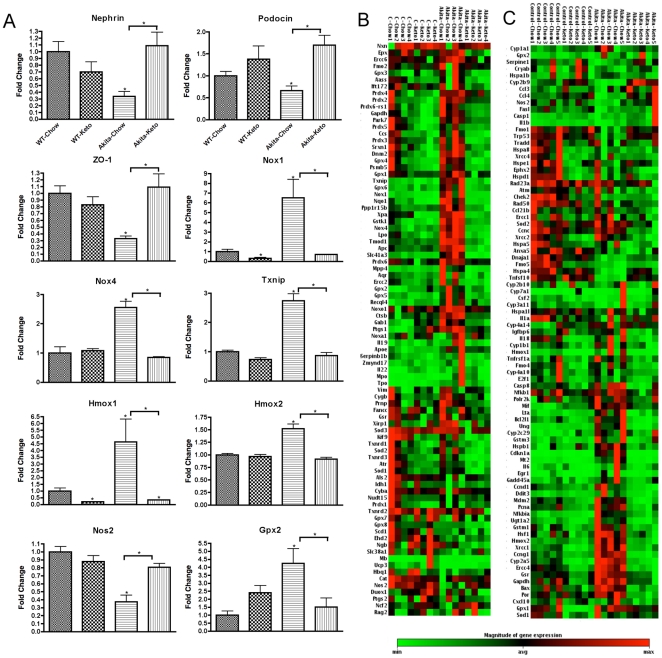
Nephropathy-related gene expression in the diabetic Akita mice is reversed by the ketogenic diet. (A) Kidney gene expression changes associated with the Akita mutation and not in the db/db diabetics that are reversed by the ketogenic diet. The pattern of reversal is particularly evident in the clusterograms form the entire (B) Oxidative Stress and Antioxidant Defense PCR Array and (C) Stress & Toxicity PCR array from SABiosciences. The clusterograms (heat maps) represent relative expression levels for all samples and all genes included on the real-time PCR arrays. Data for each gene in (A) was normalized to a panel of housekeeping transcripts and expressed as fold change compared to the WT-Chow group (except for nephrin, podocin, and zo-1 – see *Research Design and* *Methods*). Data are means ± SE (n = 4–5 for all groups). *p<0.05 (ANOVA) vs. WT-Chow group (or vs. diabetic-Chow groups as indicated by the horizontal brackets).

Consistent with the generally more robust effect of Type 1 vs. Type 2 diabetes on kidney gene expression, there were a number of genes whose expression in kidney was influenced in Akita mice but not db/db mice. Of particular interest were the inhibition of nephrin, podocin, and Z0-1, expression of which is inhibited in the streptozotocin model of Type 1 diabetes and whose inhibition is plausibly contributory to impaired function in diabetic nephropathy [25] (Figure 3A). As with the stress-induced genes, the effect of diabetes on the expression of these genes was completely reversed by the ketogenic diet, which would plausibly contribute to the reversal of nephropathy. Conversely, the induction of three genes, Nox1, Nox4, and Txnip, by diabetes plausibly contributes to pathology by producing oxidative stress [4], [23], and the complete reversal of the elevation of these genes by the ketogenic diet plausibly contributes to the reversal of neuropathy. In contrast, the elevation of Hmox1, Hmox2, and Gpx2 by diabetes probably reflects, rather than causes, oxidative stress, and the reversal of the elevation of these genes probably reflects reversal of the oxidative stress (as was probably the case in most of the genes shown in Figure 2). Although the graphs in Figure 3A were those for whose effects of diabetes were significant, the consistent pattern for other stress-related genes to be elevated by diabetes and reversed by the ketogenic diet was quite striking for both genes associated with oxidative stress (Figure 3B) and genes associated with other forms of cellular stress (Figure 3C).

**Figure 4:**
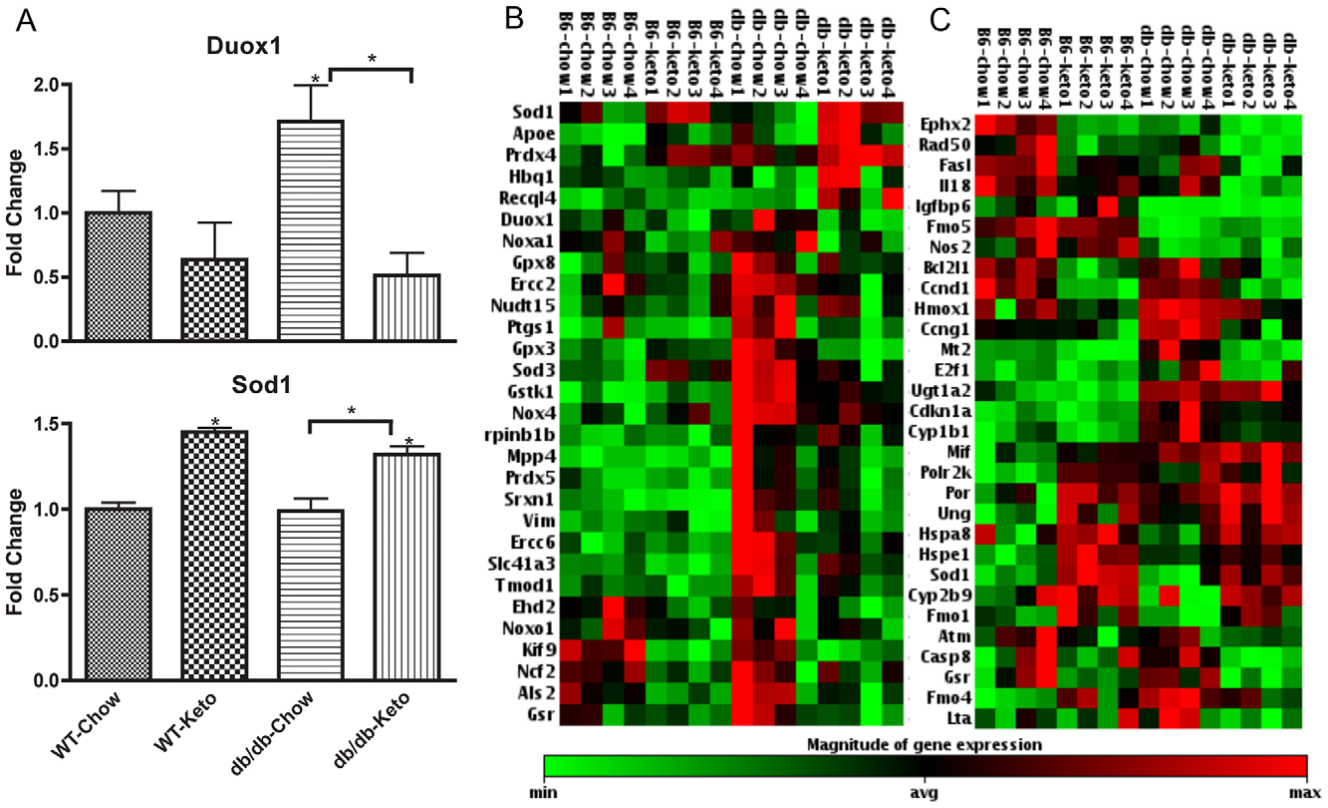
Kidney expression of duox1 and sod1 is affected by the ketogenic diet in *db/db* diabetic mice. (A) Ketogenic diet induces Sod1 expression and normalizes Duox1 expression in the *db/db* mice. The transcripts that show consistent patterns across *db/db* data sets are depicted in the clusterograms from the (B) Oxidative Stress and Antioxidant Defense PCR Array and (C) Stress & Toxicity PCR array from SABiosciences. The clusterograms (heat maps) represent relative expression levels for all samples and selected genes included on the real-time PCR arrays. Data for each gene in (A) was normalized to a panel of housekeeping transcripts and expressed as fold change compared to the WT-Chow group. Data are means ± SE (n = 4 for all groups). *p<0.05 (ANOVA) vs. WT-Chow group (or vs. diabetic-Chow groups as indicated by the horizontal bracket).

Although fewer stress-related genes were induced in kidney from db/db than Akita mice, at least one gene, Duox1, was induced in db/db but not in Akita mice (Figure 4A). This gene is a member of the NADPH oxidase gene family and thus, as with Nox1 and Nox4 in kidneys from Akita mice, its induction with diabetes plausibly contributes to the development of nephropathy, and its reversal by the ketogenic diet plausibly contributes to the reversal of pathology by the diet. In contrast, Sod1 exhibits an unusual pattern, in that it is not influenced by diabetes but was induced by the ketogenic diet in both wild-type and db/db mice (Figure 4A), suggesting that the induction of Sod1 could mediate part of the protective effect of the ketogenic diet. Consistent with the observation that effects of diabetes in db/db mice were less robust than in Akita mice, the heat-maps of stress-induced genes exhibited less robust effects of gene expression, though some induction of these genes, and reversal of the induction, was still discernable (Figure 4B, 4C).

### Partial reversal of histological evidence of pathology in diabetes

**Figure 5:**
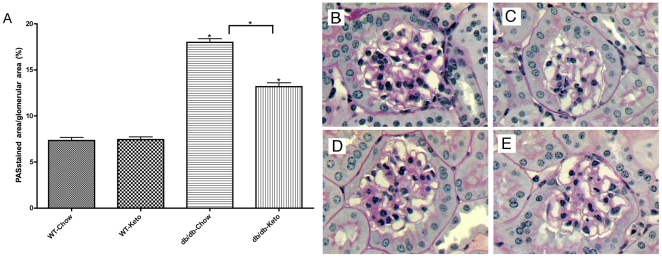
Improvement in glomerular histopathology in diabetic *db/db* mice fed the ketogenic diet. Morphometric study demonstrating the mean values of glomerular deposition of PAS positive material(A) (% of total glomerular area) in *db/db* using light microscopy in fixed kidney stained with periodic-acid-Schiff stain as described in *Research Design and* *Methods*. Images of representative glomerular histology from WT-Chow (B), WT-Keto (C), *db/db*-Chow (D), *db/db*-Keto (E). *p<0.05 (ANOVA) vs. Control groups (or vs. db/db-Chow group - horizontal bracket).

As shown in Figure 5, in contrast to albumin/creatine ratios that were completely reversed by the ketogenic diet, histological pathology (e.g., glomerular sclerosis) was only partially (though significantly) reversed by the ketogenic diet in diabetic mice. Representative glomerular histology is presented in Figure 5 (panels B–E). These conclusions were corroborated by a blind qualitative assessment of the mesangial matrix expansion rating using a scale of 0–3. These data suggest that reversal of functional and molecular markers of nephropathy occur more rapidly than reversal of histological markers of nephropathy.

### The ketone 3-OHB is cytoprotective

**Figure 6:**
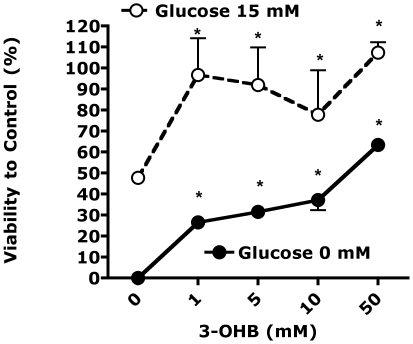
The ketone 3-beta-hyroxybutyrate (3-OHB) is cytoprotective in cells exposed to 100 uM hydrogen peroxide, at 0 or 15 mM glucose. p<0.001, ANOVA, significant effect of 3-OHB at both glucose concentrations. * p<0.05 vs. 0 3-OHB. Viability determined by a colorimetric (CCK-8; Dojin Laboratories, Kumamoto, Japan).

To further assess potential mechanisms mediating the protective effects of the ketogenic diet, and since glucose toxicity in diabetes is thought to be mediated by glucose-induced oxidative stress, we assessed if the ketone 3-OHB would protect cells from oxidative stress enhanced by either high or low glucose. As shown in Figure 6, 3-OHB produced a dose-responsive cytoprotective effect at both elevated and reduced glucose.

## Discussion

In contrast to previous studies in which good glucose control prevented, but did not reverse, nephropathy in a model of Type 1 diabetes [2], in the present studies the ketogenic diet reversed nephropathy, as reflected by albumin/creatinine ratios, after it had developed in models of both Type 1 and Type 2 diabetes. The reversal of functional nephropathy was associated with robust normalization of expression of genes induced by oxidative and other forms of stress. In contrast to the complete reversal of nephropathy as reflected by albuminuria and gene expression, histological evidence of nephropathy was only partially reversed in the model for Type 2 diabetes (kidneys from the Akita mice were not available for histological analysis). This suggests, perhaps not surprisingly, that functional and molecular aspects of nephropathy reverse more quickly than morphological aspects of diabetic nephropathy.

The present study also confirmed and greatly extended the number of molecular markers for diabetic nephropathy, especially for genes whose expression in kidney is induced in models of both Type 1 and Type 2 diabetes. The gene most robustly induced in both forms of diabetes was Cdkn1a, also known as p21 (Figure 2A). Cdkn1a was induced over 20-fold in Akita mice though only about 4-fold in db/db mice, consistent with the generally more robust effect of diabetes on gene expression in Akita mice compared to db/db mice, though interestingly albuminuria was greater in db/db mice than in Akita mice. Although induction of p21 has been reported in a rat model of Type 1 diabetic nephropathy [26], the functional significance of this observation is unclear. P21 is induced by a number of cellular stressors and generally appears to play a role in ameliorating stresses, including DNA damage [27], so it seems likely that in the present studies the induction of p21 and its reversal by the ketogenic diet reflects pathological processes rather than mediating the reparative effects of the ketogenic diet. Indeed, this appears to be the case for the vast majority of the genes whose expression was induced in both forms of diabetic nephropathy and whose induction was reversed by the ketogenic diet. Possible exceptions to this pattern are Nox1, Nox4, Txnip, and Duox1, whose induction all plausibly contribute to the development of oxidative stress [4], [23], and thus whose inhibition by the ketogenic diet all plausibly contribute to the restorative effects of the ketogenic diet. Similarly, Casp-8 was inhibited by the ketogenic diet in diabetic mice, and it has been reported that in *db/db* mice decreased caspase-8 activity correlates with decreased progression of diabetic nephropathy with moderate exercise [28], and podocyte apoptosis appears to be a feature of advanced diabetic nephropathy [26]. More directly reflective of nephropathy are the inhibition of nephrin, podocin, and ZO-1, all of which contribute to normal kidney function and restoration of which by the ketogenic diet plausibly reflects restoration of normal kidney function.

We also note that in contrast to the robust weight loss that the ketogenic diet produces in diet-induced obesity [13], the ketogenic diet increased, rather than decreased, body weight in db/db mice (Table 1). Furthermore, weight gain occurred even though caloric intake decreased. It has similarly been reported that the ketogenic diet does not reduce body weight in leptin-deficient ob/ob mice [29], which we have also observed (Mastaitis, unpublished). Thus the weight gain observed in db/db mice, in contrast to the weight loss in wild-type mice, is presumably due to the fact leptin signaling is required for the reduction in body weight by the ketogenic diet, and the weight gain despite reduced caloric intake presumably reflects reduced metabolic rate. It will be of interest to determine which cell types mediate this requirement for leptin in weight loss by the ketogenic diet.

A key issue raised but not resolved in the present study is the mechanism by which the ketogenic diet reverses nephropathy and gene expression profiles associated with nephropathy. One potential mechanism is that the reversal was simply due to reduction in blood glucose. However, since previous studies demonstrated that good glucose control prevented, but did not reverse, diabetic nephropathy [2], and since diabetic complications are thought to be caused by increased cellular metabolism of glucose [30], we hypothesize that at least part of the restorative effect was mediated by reduction of glucose metabolism. This hypothesis is supported by several lines of evidence. First, the ketogenic diet appears to reduce the frequency of epileptic seizures by reducing glucose metabolism [11], [12]. Second, molecular responses to the ketogenic diet indicates a re-routing of cellular metabolism away from glucose utilization and toward the use of alternative fuels [13], [14]. Finally, we have shown that ketone 3-OHB blocks molecular effects of glucose [9]. We therefore hypothesize that the ketogenic diet reverses diabetic nephropathy by raising blood levels of 3-OHB which subsequently reduce glucose metabolism in at least some tissues including kidney. Since ketones and the ketogenic diet are neuroprotective in a wide range of conditions [31], a phenomenon we have corroborated in the present study (Figure 6), it seems highly likely that the ketogenic diet will be protective in diabetic neuropathy and possibly retinopathy as well.

Although the present studies represent a proof of principle that pathologies produced by diabetes can be reversed by a simple dietary manipulation, many issues remain to be resolved before clinical application can be considered. First, the ketogenic diet is probably too extreme for chronic use in adult patients, and indeed may produce untoward iatrogenic effects. On the other hand, based on hysteretic mechanisms observed with the lac operon [7], [8], it is plausible that only transient exposure to the diet will effectively reverse the gene expression profile and thus in effect “reset” the pathological process. If so it is plausible that only a sustainable transient exposure to the diet may be needed to produce persistent reversal of pathologies associated with diabetes. Furthermore, if the mechanism by which the diet produces its protective effects is in fact the elevation of blood 3-bOHB, it is possible that a pharmacological intervention that mimics these effects might be sufficient to reverse pathology. Although we have not yet been successful in chronically elevating plasma 3-bOHB by chronic mini-pump infusion, dietary supplementation remains a possibility, including supplementation with ketogenic derivatives [32]. We have also demonstrated that chronic administration of the structurally similar molecular butyrate (differing only by a single oxygen atom) mimics many of the protective effects of dietary restriction through a mechanism dependent on the transcription factor CBP [33], so assessing the efficacy of this or related molecules in reversing pathologies due to diabetes would also be of great interest.

## References

[1] Crofford OB (1995). Diabetes control and complications.. Annu Rev Med.

[2] Kowluru RA, Abbas SN, Odenbach S (2004). Reversal of hyperglycemia and diabetic nephropathy: effect of reinstitution of good metabolic control on oxidative stress in the kidney of diabetic rats.. J Diabetes Complications.

[3] Mobbs CV, Mastaitis JW, Zhang M, Isoda F, Cheng H (2007). Secrets of the lac operon. Glucose hysteresis as a mechanism in dietary restriction, aging and disease.. Interdiscip Top Gerontol.

[4] Hamada Y, Fukagawa M (2007). A possible role of thioredoxin interacting protein in the pathogenesis of streptozotocin-induced diabetic nephropathy.. Kobe J Med Sci.

[5] El-Osta A, Brasacchio D, Yao D, Pocai A, Jones PL (2008). Transient high glucose causes persistent epigenetic changes and altered gene expression during subsequent normoglycemia.. J Exp Med.

[6] Tonna S, El-Osta A, Cooper ME, Tikellis C (2010). Metabolic memory and diabetic nephropathy: potential role for epigenetic mechanisms.. Nat Rev Nephrol.

[7] Laurent M, Charvin G, Guespin-Michel J (2005). Bistability and hysteresis in epigenetic regulation of the lactose operon. Since Delbruck, a long series of ignored models.. Cell Mol Biol (Noisy-le-grand).

[8] Ozbudak EM, Thattai M, Lim HN, Shraiman BI, Van Oudenaarden A (2004). Multistability in the lactose utilization network of Escherichia coli.. Nature.

[9] Cheng H, Isoda F, Belsham DD, Mobbs CV (2008). Inhibition of agouti-related peptide expression by glucose in a clonal hypothalamic neuronal cell line is mediated by glycolysis, not oxidative phosphorylation.. Endocrinology.

[10] Bailey EE, Pfeifer HH, Thiele EA (2005). The use of diet in the treatment of epilepsy.. Epilepsy Behav.

[11] Ma W, Berg J, Yellen G (2007). Ketogenic diet metabolites reduce firing in central neurons by opening K(ATP) channels.. J Neurosci.

[12] Garriga-Canut M, Schoenike B, Qazi R, Bergendahl K, Daley TJ (2006). 2-Deoxy-D-glucose reduces epilepsy progression by NRSF-CtBP-dependent metabolic regulation of chromatin structure.. Nat Neurosci.

[13] Kennedy AR, Pissios P, Otu H, Xue B, Asakura K (2007). A high-fat, ketogenic diet induces a unique metabolic state in mice.. Am J Physiol Endocrinol Metab.

[14] Badman M, Pissios P, Kennedy AR, Koukos G, Flier J (2007). Hepatic fibroblast growth factor 21 is regulated by PPARalpha and is a key mediator of hepatic lipid metabolism in ketotic states.. Cell Metab.

[15] Pelsman A, Hoyo-Vadillo C, Gudasheva TA, Seredenin SB, Ostrovskaya RU (2003). GVS-111 prevents oxidative damage and apoptosis in normal and Down's syndrome human cortical neurons.. Int J Dev Neurosci.

[16] Gum ET, Swanson RA, Alano C, Liu J, Hong S (2004). Human serum albumin and its N-terminal tetrapeptide (DAHK) block oxidant-induced neuronal death.. Stroke.

[17] Osakada F, Kawato Y, Kume T, Katsuki H, Sugimoto H (2004). Serofendic acid, a sulfur-containing diterpenoid derived from fetal calf serum, attenuates reactive oxygen species-induced oxidative stress in cultured striatal neurons.. J Pharmacol Exp Ther.

[18] Lee Y, Aono M, Laskowitz D, Warner DS, Pearlstein RD (2004). Apolipoprotein E protects against oxidative stress in mixed neuronal-glial cell cultures by reducing glutamate toxicity.. Neurochem Int.

[19] Mercer LD, Kelly BL, Horne MK, Beart PM (2005). Dietary polyphenols protect dopamine neurons from oxidative insults and apoptosis: investigations in primary rat mesencephalic cultures.. Biochem Pharmacol.

[20] Grant MM, Barber VS, Griffiths HR (2005). The presence of ascorbate induces expression of brain derived neurotrophic factor in SH-SY5Y neuroblastoma cells after peroxide insult, which is associated with increased survival.. Proteomics.

[21] Ho L, Qin W, Pompl PN, Xiang Z, Wang J (2004). Diet-induced insulin resistance promotes amyloidosis in a transgenic mouse model of Alzheimer's disease.. Faseb J.

[22] Wang J, Ho L, Qin W, Rocher AB, Seror I (2005). Caloric restriction attenuates beta-amyloid neuropathology in a mouse model of Alzheimer's disease.. Faseb J.

[23] Susztak K, Raff AC, Schiffer M, Bottinger EP (2006). Glucose-induced reactive oxygen species cause apoptosis of podocytes and podocyte depletion at the onset of diabetic nephropathy.. Diabetes.

[24] Kojima S, Asakawa A, Amitani H, Sakoguchi T, Ueno N (2009). Central leptin gene therapy, a substitute for insulin therapy to ameliorate hyperglycemia and hyperphagia, and promote survival in insulin-deficient diabetic mice.. Peptides.

[25] Barutta F, Corbelli A, Mastrocola R, Gambino R, Di Marzo V (2010). Cannabinoid receptor 1 blockade ameliorates albuminuria in experimental diabetic nephropathy.. Diabetes.

[26] Menini S, Iacobini C, Oddi G, Ricci C, Simonelli P (2007). Increased glomerular cell (podocyte) apoptosis in rats with streptozotocin-induced diabetes mellitus: role in the development of diabetic glomerular disease.. Diabetologia.

[27] Cazzalini O, Scovassi AI, Savio M, Stivala LA, Prosperi E (2010). Multiple roles of the cell cycle inhibitor p21(CDKN1A) in the DNA damage response.. Mutat Res.

[28] Ghosh S, Khazaei M, Moien-Afshari F, Ang LS, Granville DJ (2009). Moderate exercise attenuates caspase-3 activity, oxidative stress, and inhibits progression of diabetic renal disease in db/db mice.. Am J Physiol Renal Physiol.

[29] Badman MK, Kennedy AR, Adams AC, Pissios P, Maratos-Flier E (2009). A Very Low Carbohydrate Ketogenic Diet Improves Glucose Tolerance in ob/ob Mice Independent of Weight Loss.. Am J Physiol Endocrinol Metab.

[30] Brownlee M (2001). Biochemistry and molecular cell biology of diabetic complications.. Nature.

[31] Maalouf M, Rho JM, Mattson MP (2009). The neuroprotective properties of calorie restriction, the ketogenic diet, and ketone bodies.. Brain Res Rev.

[32] Smith SL, Heal DJ, Martin KF (2005). KTX 0101: a potential metabolic approach to cytoprotection in major surgery and neurological disorders.. CNS Drug Rev.

[33] Zhang M, Poplawski M, Yen K, Cheng H, Bloss E (2009). Role of CBP and SATB-1 in aging, dietary restriction, and insulin-like signaling.. PLoS Biol.

